# The effectiveness of an emotional intelligence intervention on Lebanese adolescent mental health: A mixed‐method study with emotional diary integration

**DOI:** 10.1111/bjdp.70061

**Published:** 2026-07-31

**Authors:** M. J. Sanchez‐Ruiz, S. El‐Hajj, J. M. Gimenez‐Lozano, A. Hernández‐Saguar, M. El Khoury‐Malhame, R. Doumit, K. Petrides, M. Checa‐Romero

**Affiliations:** ^1^ Department of Personality Psychology, Assessment, and Psychological Treatment, School of Psychology National University of Distance Education (UNED) Madrid Spain; ^2^ Department of English and Creative Arts, School of Arts and Sciences Lebanese American University Beirut Lebanon; ^3^ Department of Psychology ESCUNI Madrid Spain; ^4^ Department of Psychology Valencian International University Valencia Spain; ^5^ Department of Psychology and Education, School of Arts and Sciences Lebanese American University Beirut Lebanon; ^6^ Alice Ramez Chaghoury School of Nursing Lebanese American University Beirut Lebanon; ^7^ Department of Psychology and Psychometrics University College of London London UK; ^8^ Department of Educational Science Universidad de Alcalá Madrid Spain

**Keywords:** coping, emotional diaries, Lebanon, mixed‐method, trait emotional intelligence, vulnerable adolescence

## Abstract

Adolescents in Lebanon face socioeconomic and political stressors that undermine socioemotional development and mental health. This study evaluated the Yes to Emotions (YEY) programme, a culturally adapted 11‐session intervention to enhance trait Emotional Intelligence (trait EI) and mental health. The sample comprised 712 adolescents (326 girls) aged 14–21 years from public schools allocated to either the YEY intervention (with voluntary diary completion) or a waitlist comparison condition. A mixed‐methods design combined self‐report and diary data. Results showed higher trait EI, intrapersonal emotional competencies, adaptive coping and reduced maladaptive coping and depressive symptoms. Effect sizes were larger in the diary group, whereas the non‐diary group showed more modest gains and often did not differ from the waitlist comparison group. Qualitative findings illustrated the quantitative results, illustrating improvements in emotion regulation, self‐esteem, empathy and interpersonal relationships. Findings support EI training and the potential value of emotional diaries in high‐adversity contexts.

## INTRODUCTION

Adolescence, as a pivotal stage of rapid neurobiological, emotional and social development, makes individuals especially sensitive to environmental stressors (Ford & Gross, 2019; Gross, 2002). In Lebanon, ongoing political instability, economic collapse and social unrest have heightened adolescents' vulnerability to psychological distress (Khalil et al., 2025; Nasr et al., 2022). These overlapping crises, together with social inequality and limited access to education and healthcare, expose youth to chronic adversity that can hinder emotional development and mental health (Maalouf et al., 2022). Identifying protective factors that foster adaptive functioning is therefore crucial, with emotional competencies emerging as key to promoting well‐being, resilience and positive adjustment amid instability (McLoughlin & Kristjánsson, 2025).

Despite this need, few interventions targeting emotional development and mental health are available. One psychological construct of particular relevance in this context is trait Emotional Intelligence (trait EI), which refers to a constellation of emotional self‐perceptions measured through questionnaires and rating scales (Petrides et al., 2007). It is conceptualized as a personality‐based construct concerning how people perceive, interpret and manage emotional information in daily life (Petrides, 2009). Trait EI has gained considerable attention because of its predictive value for a wide range of outcomes, including academic engagement and performance, physical and mental health, life satisfaction, relationship quality and other indicators of well‐being (see Petrides et al., 2016 for a review).

The purpose of this study is to evaluate the effectiveness of the *Yes to Emotions (YEY)* programme, a culturally adapted, youth‐led intervention designed to enhance trait EI among at‐risk adolescents in Lebanon. In addition to assessing changes in emotional competences, we examined the potential contribution of integrating emotional diaries (ED) as a reflective tool to deepen emotional learning.

### Trait EI and relevance

A growing body of research has demonstrated that trait EI is positively associated with a range of desirable life outcomes, including academic achievement, quality of interpersonal relationships, professional success and both physical and mental health (Andrei & Petrides, 2013; González‐Yubero et al., 2025). These associations are consistent across cultures despite contextual variation in emotional development (Mancini et al., 2024; Yang et al., 2025). Trait EI is organized into four interrelated dimensions: (i) well‐being, including self‐esteem, optimism and happiness; (ii) self‐control, including emotion regulation, stress management and impulse control; (iii) emotionality, including emotion perception, emotion expression, empathy and relationships; and (iv) sociability, including assertiveness, social awareness and emotion management. This multidimensional structure provides a solid framework for understanding how emotional traits influence psychological outcomes across different social and cultural settings.

Trait EI has been shown to contribute to emotional development from early childhood onwards (Solih et al., 2024). Although relatively stable, it remains sufficiently malleable to be strengthened through experience (Sanchez‐Ruiz & van der Linden, 2024). Personality traits demonstrate moderate plasticity across development (Roberts et al., 2006), particularly during adolescence, a period characterized by heightened socioemotional and neurodevelopmental flexibility (Soto & Tackett, 2015). Individuals with higher trait EI tend to adopt more adaptive coping strategies, including active coping (Sáez‐Delgado et al., 2023) and planning (Macías‐Espinoza et al., 2022), which facilitate adjustment to everyday challenges, particularly in high‐stress environments.

Trait EI has also been positively associated with adaptive coping, peer‐rated social competence and psychological well‐being and negatively associated with depression, somatic complaints and maladaptive coping (Mavroveli et al., 2007). In the Lebanese context, higher trait EI has likewise been associated with better emotional regulation, lower anxiety and depression and greater psychological well‐being (El Khodary et al., 2025), highlighting its potential as a protective resource in contexts of chronic adversity. Accordingly, trait EI can be enhanced through structured emotional learning interventions targeting emotional awareness, regulation and interpersonal functioning (Hodzic et al., 2018; Kotsou et al., 2011; Nelis et al., 2009). Reviews of trait EI interventions report average increases of approximately 12% in self‐reported trait EI, with effects lasting up to 1 year (Mikolajczak & Peña‐Sarrionandia, 2015). These improvements have also been associated with enhanced well‐being, interpersonal relationships, physical health and reduced psychological distress (Kotsou et al., 2011; Petrides et al., 2016). Among adolescents, trait EI is particularly relevant in educational settings, where it predicts better school adjustment, prosocial behaviour, academic engagement and performance, fewer unauthorized absences and rule violations (e.g., Mavroveli & Sánchez‐Ruiz, 2011; Petrides et al., 2004, 2018). A recent systematic review further supports its positive association with adolescent well‐being (Mancini et al., 2024).

### Trait EI in SEL in education: From frameworks to outcomes of SEL programmes

Social emotional learning (SEL) is an evidence‐based, school‐based pedagogical approach that aims to develop emotional and social competencies essential for personal growth, academic achievement and lifelong well‐being (Hachem et al., 2022; Kim et al., 2024).

The integration of trait EI into educational interventions has become a key area of interest, particularly through SEL frameworks (Trish, 2023). One of the most widely adopted models is CASEL (Collaborative for Academic, Social and Emotional Learning; Pérez‐González & Qualter, 2018), which identifies five core competencies: self‐awareness, self‐management, social awareness, relationship skills and responsible decision‐making (Weissberg et al., 2015). These competencies promote emotion regulation, healthier relationships, responsible decision‐making and supportive learning environments (Ramirez et al., 2021; Ulla & Poom‐Valickis, 2023).

Evidence consistently shows that SEL improves mental health, classroom behaviour, academic performance, socio‐emotional development and long‐term well‐being, with particularly strong effects in teacher‐delivered programmes and among marginalized students (Cipriano et al., 2024; Lovett et al., 2024; Mangrio et al., 2024; Shi & Cheung, 2024).

### The ‘Yes to Emotions’ (YEY) project: A SEL‐based intervention for vulnerable adolescents in Lebanon

The current study is part of the YEY project, a structured SEL‐based intervention specifically designed for adolescents experiencing social vulnerability in Lebanon. YEY targets adolescents exposed to poverty, family instability, economic collapse and social marginalization, all of which characterize the current Lebanese context (Sanchez‐Ruiz et al., 2025). In recent years, prolonged sociopolitical instability, the Beirut port explosion, the COVID‐19 pandemic and the influx of Syrian refugees have further intensified these adversities, contributing to high levels of anxiety, depression and post‐traumatic stress among Lebanese youth (El Hajj, 2023a, 2023b; El Khoury‐Malhame, Harajli, et al., 2024; Farran, 2021; Maalouf et al., 2022). Despite these challenges, trait EI has emerged as a potential protective factor promoting adaptive coping (Sanchez‐Ruiz et al., 2021).

The intervention focuses on strengthening emotional competencies through a developmentally appropriate, participatory approach rooted in youth‐led discussions and reflective exercises. YEY is grounded in the Profile of Emotional Competence (PEC) model (Mikolajczak et al., 2014), which encompasses five emotional competencies: (1) Identification: recognizing and labelling one's own emotions and those of others; (2) Comprehension: understanding emotional causes and consequences; (3) Expression: communicating emotions clearly and appropriately; (4) Regulation: managing emotions functionally and adaptively; (5) Utilization: using emotions to support reasoning, decision‐making and action (Brasseur et al., 2013). Cece et al. (2023) implemented an EI training programme for adolescents, using the PEC to assess outcomes, and reported significant improvements in emotional competencies and psychosocial adjustment.

The programme develops the five emotional competencies of the PEC, which are associated with resilience, mental health, academic adjustment and social integration among vulnerable adolescents (Zeidner et al., 2012). Through youth‐led discussions and reflective exercises, participants explore their emotional experiences in a supportive environment that encourages emotional awareness, communication and regulation (Warraitch et al., 2024). By creating a supportive and non‐judgemental environment, the programme encourages young people to openly discuss emotions, understand the impact of their emotional responses and learn effective strategies for emotional regulation.

After each session, participants completed emotional diaries documenting emotional triggers and responses. This reflective activity encouraged them to apply the five PEC competencies to everyday experiences, thereby reinforcing emotional learning (Baikie & Wilhelm, 2005; Pennebaker, 1997; Travagin et al., 2015).

### The present study

Adolescence represents a sensitive developmental period in which emotional competencies shape psychological adjustment and resilience, whereas exposure to adversity may have lasting effects on emotional regulation, mental health and psychosocial functioning (Banati et al., 2025; Irshad & Lone, 2025; Masten & Cicchetti, 2010; Sawyer et al., 2012). The socioemotional competencies targeted by YEY align with current SEL models and are recognized as protective factors for adolescent mental health, adaptive coping and social integration, particularly among vulnerable youth (Chelouche‐Dwek & Fonagy, 2024; El Khoury‐Malhame, Bou Malhab, et al., 2024; Taylor et al., 2017). However, most emotional education interventions have been developed in Western or high‐income contexts, leaving limited evidence on whether SEL interventions can strengthen trait EI and coping among adolescents in settings such as Lebanon (Karam et al., 2025).

The current study addresses this societal and research gap by evaluating the effectiveness of the culturally adapted YEY programme, based on the PEC model (Mikolajczak et al., 2014) and funded by Grand Challenges Canada—Global Mental Health (Grand Challenges Canada, 2021). Using a mixed‐methods design that combines standardized measures of trait EI, coping and psychological well‐being with qualitative data from emotional diaries, the study examines whether YEY enhances emotional competencies, adaptive coping and well‐being among adolescents living in conditions of adversity.

## METHODS

### Research design

This study employed a quasi‐experimental pre/post‐test mixed‐methods design integrating quantitative and qualitative data collection and analysis. The design included one waitlist comparison group and one intervention condition, within which participants could choose to complete emotional diaries (ED), resulting in three groups: (1) waitlist comparison, (2) YEY without ED, and (3) YEY with ED. All groups were included in the quantitative analyses, whereas qualitative data were derived exclusively from participants who completed ED. This design enabled evaluation of both the overall effectiveness of YEY and the potential added value of ED, which had shown promising effects in pilot work.

The intervention received approval from the Lebanese Ministry of Education and Higher Education (MEHE) and ethical clearance from the Lebanese American University Institutional Review Board (LAU.SAS.MJ1.28). Prior to implementation, youth leaders received training in emotional competencies and participatory pedagogy. The programme and assessment tools were piloted in a Lebanese semi‐private school, and feedback from students and facilitators informed refinements to programme activities and measurement tools to improve cultural relevance, clarity and participant engagement.

Following ethical approval, informed parental consent and adolescent assent were obtained before data collection commenced. Participants completed standardized assessments before the intervention, immediately afterwards, and at 6‐month follow‐up, although follow‐up findings are beyond the scope of the present study and will be reported separately. The intervention consisted of 11 weekly sessions organized into five modules targeting emotional identification, understanding, expression, regulation and utilization (Brasseur et al., 2013). Activities included mindfulness, emotional journaling, visualization, discussions, role play, take‐home tasks and self‐reflection delivered through youth‐led participatory sessions consistent with SEL recommendations (Pérez‐González & Qualter, 2018).

### Participants

As shown in Table 1, the sample comprised 712 high school students aged 14–21 years (*M* = 16.46, SD = 1.25) recruited from three Lebanese public schools selected from a list provided by the Ministry of Education and Higher Education (MEHE). All schools followed the national curriculum and were selected from different regions while excluding single‐sex schools and ensuring a sufficient sample size for group comparisons. Schools, rather than individuals, were allocated to study conditions, with two assigned to the YEY intervention and one serving as the waitlist comparison group. Of the initial sample, 610 participants completed the post‐intervention assessment, of whom 53.4% were female (*N* = 326).

**TABLE 1:** Descriptive statistics of participant characteristics and key study variables.

	Categories	*N*	*M* (SD) or %	Min	Max
Gender	Women	326	53.4%		
	Men	271	44.4%		
	Non‐binary	13	2.1%		
Age		568	16.42 (1.24)	14.00	21.00
Paternal employment status	Employed	526	91.2%		
	Unemployed	51	8.8%		
Maternal employment status	Employed	164	27.5%		
	Unemployed	462	72.5%		
Difficulty paying bills	Yes	453	81.2%		
	No	105	18.8%		
Study groups	WCG	359	50.4%		
	Intervention with ED	144	20.2%		
	Intervention without ED	209	29.4%		
Anxiety (GAD‐7)		543	9.63 (5.73)	0.00	21.00
Global EC (PEC)		543	3.28 (0.45)	1.97	4.49
Interpersonal EC (PEC)		543	3.42 (0.51)	1.88	4.84
Intrapersonal EC (PEC)		543	3.14 (0.56)	1.38	4.50
Depression (PHQ‐9)		493	11.28 (7.06)	0.00	32.00
Global trait EI (TEIQue‐SF)		279	4.44 (0.89)	1.97	6.67
Well‐being		426	4.90 (1.38)	1.00	7.00
Self‐control		429	3.94 (1.25)	1.00	7.00
Emotionality		410	4.17 (0.93)	1.75	6.88
Sociability		419	4.80 (1.05)	1.17	7.00
Well‐being (WHO‐5)		542	10.05 (5.49)	0.00	25.00
Adaptive coping (brief‐COPE)		536	22.74 (4.59)	9.00	32.00
Maladaptive coping (Brief‐COPE)		536	14.62 (4.46)	8.00	29.00
Instrumental coping (Brief‐COPE)		536	9.60 (2.92)	4.00	16.00

Abbreviations: EC, emotional competencies; ED, intervention group with emotional diary; no ED, intervention group without emotional diary; WCG, waitlist comparison group.

Within the intervention schools, participation in the emotional diary (ED) component was voluntary, resulting in three groups: YEY + ED (20.2%, *n* = 144), YEY only (29.4%, *n* = 209) and the waitlist comparison group (50.4%, *n* = 359). Because participation in ED was voluntary, potential self‐selection bias was assessed by comparing both intervention groups on the main outcome variables. Significant baseline differences were observed only for global emotional competence (*p* = .036), interpersonal emotional competence (*p* = .044) and well‐being (*p* = .011), indicating broad comparability across the remaining variables, although self‐selection cannot be completely ruled out (Van de Groep et al., 2020). Participants also reported parental employment (91.2% of fathers and 27.5% of mothers employed) and moderate financial strain (*M* = 5.28, SD = 1.94), with 81.2% reporting moderate‐to‐severe difficulty paying household bills.

## STUDY 1: QUANTITATIVE PHASE

### Study 1: Method

#### Data collection

Quantitative data were collected through standardized self‐report questionnaires administered at two time points: pre‐intervention (baseline) and immediately after completion of the programme (post‐intervention). Data collection was conducted in person at the schools, in 35–40 min group sessions scheduled within the hours assigned to the project. Students completed the questionnaires individually in supervised classroom settings, and trained research staff were present to provide instructions and address any questions. The same procedure was applied across the three participating schools.

#### Measures

##### Trait EI questionnaire for adolescents—Short form

The short form of the Trait EI Questionnaire for Adolescents (TEIQue‐ASF; Petrides et al., 2006) evaluates overall trait EI through 30 statements (e.g., ‘I usually find it difficult to regulate my emotions’), using a 7‐point Likert scale ranging from 1 (‘Completely Disagree’) to 7 (‘Completely Agree’). The TEIQue has demonstrated robust internal consistency in adolescent samples internationally (Nguyen et al., 2025) and also in young adults in Lebanon (Sanchez‐Ruiz et al., 2021, 2026). In our sample, internal consistency was .88 for the global scale, .83 for wellbeing, .71 for self‐control, .46 for emotionality and .63 for sociability.

##### Profile of emotional competence

The Profile of Emotional Competence (PEC; Brasseur et al., 2013) assesses emotional competence across both intrapersonal and interpersonal domains. Each domain is evaluated through five key emotional skills: emotion identification, comprehension, expression, regulation and utilization. Participants responded to 50 items (e.g., ‘As my emotions arise, I don't understand where they come from’) on a 5‐point Likert‐type scale ranging from 1 (‘Does not describe me at all’) to 5 (‘Describes me very well’). The Arabic version of the PEC demonstrated good reliability for the global score (α = .86), intrapersonal (α = .75) and interpersonal dimensions (α = .68; Aouani et al., 2019). In our study, the internal consistency was .84 for the global scale and .79 for both the intrapersonal and interpersonal emotional competence subscales.

##### Brief COPE inventory

The Brief COPE (Carver, 1997) is a self‐report measure assessing coping responses. It includes 28 items grouped into 14 subscales, each consisting of two items, encompassing adaptive and maladaptive coping strategies. Participants rated their frequency of use of different coping strategies (e.g., ‘I've been accepting the reality of the fact that it has happened’) *on* a 4‐point scale ranging from 1 (‘I haven't been doing this at all’) to 4 (‘I've been doing this a lot’). The instrument has demonstrated solid psychometric properties and has been validated in Arabic‐speaking populations (e.g., Alghamdi & Holland, 2020). In our sample, internal consistency ranged from .60 to .77.

##### World Health Organization well‐being index (WHO‐5)

The WHO‐5 measures subjective emotional well‐being over the previous 2 weeks. Participants rated five items (e.g., ‘I have felt cheerful and in good spirits’) on a 6‐point Likert scale from 0 (‘At no time’) to 5 (‘All of the time’). The WHO‐5 has been translated and validated in adult Arabic‐speaking populations (Sibai et al., 2009) and Lebanese adolescents (Doumit et al., 2025). In our sample, internal consistency was .81.

##### Generalized anxiety disorder scale (GAD‐7)

The GAD‐7 (Spitzer et al., 2006) is a brief self‐administered instrument that assesses anxiety symptoms over the past 2 weeks. Respondents rated how often they experienced seven anxiety‐related symptoms (e.g., ‘Feeling nervous, anxious, or on edge’) on a 4‐point Likert scale from 0 (‘Not at all’) to 3 (‘Nearly every day’). The scale has demonstrated good internal consistency in English‐speaking populations (α = .79–.92; Spitzer et al., 2006) and has been validated in Lebanese Arabic‐speaking adults (Sawaya et al., 2021) and adolescents (El Khoury‐Malhame et al., 2025). In our sample, internal consistency was .88.

##### Patient health questionnaire (PHQ‐9)

The PHQ‐9 (Kroenke et al., 2001) is a widely used instrument for assessing depressive symptomatology over the preceding 2 weeks. Each of the nine items corresponds to one of the DSM‐IV diagnostic criteria for depression (e.g., ‘Little interest or pleasure in doing things’). Responses are recorded on a 4‐point Likert scale from 0 (‘Not at all’) to 3 (‘Nearly every day’). The PHQ‐9 has demonstrated strong internal consistency (α = .82; Rehman et al., 2022) and has been validated in Lebanese samples (Dagher et al., 2023). In our sample, Cronbach's alpha was .86.

#### Statistical analysis

To assess the quantitative data, we examined the effects of the YEY intervention on trait EI (assessed through two validated instruments), coping strategies, psychological well‐being, anxiety symptoms and depressive symptoms. Mixed‐design ANOVAs were conducted to examine between‐group effects (intervention with ED, intervention without ED and waitlist comparison group) and within‐subject changes across two time points (pre‐ and post‐intervention). Assumptions of normality and homogeneity of variances were assessed using Shapiro–Wilk and Kolmogorov–Smirnov tests, Q–Q plots, Levene's test and boxplot inspection. Outliers were considered naturally occurring cases and were therefore retained in the analyses. Baseline equivalence and exploratory moderation analyses for age and gender were also examined; detailed results are reported in the Supporting Information. Due to unequal group sizes and assumption violations in some variables, robust mixed‐design ANOVAs using 20% trimmed means were conducted with the bwtrim function from the WRS2 package in R, as it provides more reliable estimates under such conditions (Field, 2024). For variables meeting parametric assumptions, standard ANOVAs were conducted.

Effect sizes were computed following Cohen's (1988) guidelines and recommendations for standardized effect reporting (Lakens, 2013). Cohen's *d* was used for baseline comparisons, whereas Morris's (2008) adjusted *d* was used to estimate between‐group intervention effects in the pretest–posttest design, accounting for pretest scores. As bwtrim does not provide pairwise post hoc comparisons, time‐based changes in robust analyses were interpreted through effect sizes. Analyses were conducted in SPSS (version 29) and R (version 4.4.3).

### Study 1: Results

Overall, the ED group showed the most consistent pattern of improvement across emotional competence, trait EI, coping and depressive symptoms. By contrast, the intervention group without diaries showed more modest gains, and for some outcomes, its change was small or did not clearly differ from that of the waitlist comparison group.

Baseline comparisons and exploratory moderation analyses are provided in the Supporting Information. In brief, no consistent moderation effects were found for age or gender. Comparisons between the two intervention groups further indicated that most pre‐intervention differences were non‐significant, and the few significant differences observed were small to moderate in magnitude (all *d* ≤ 0.50).

#### Trait EI variables

As shown in Table 2 and illustrated in Figure 1 (Supporting Information), a–n, the analysis revealed significant interaction effects for multiple EI measures depending on treatment condition. For global emotional competence (PEC; Figure 1a), a decrease was observed in the waitlist comparison group from pre‐ to post‐intervention, while scores increased in both intervention groups (more notably in the ED group). The ANOVA indicated a significant interaction effect (*F*_(2,195.73)_ = 20.50, *p* < .001). Effect sizes showed a large effect for the ED group versus the waitlist comparison group (*d* = 1.08), small for non‐ED versus the waitlist comparison group (*d* = 0.43) and moderate‐to‐large between intervention groups (*d* = −0.78), favouring ED.

**TABLE 2:** Effects of the intervention on all outcome measures: Mixed‐design ANOVA and effect sizes.

Measure	Group	*N*	Mean (SD)		Within‐subjects		Between‐group	Effect size	
			Pre	Post	Time	Time*group		Groups	*d*
Adaptive coping (Brief‐COPE)	WCG	264	23.40 (2.73)	22.00 (3.15)	*F*_(1,533)_ = 2.21, *p* = .137	*F*_(2,533)_ = 16.26, *p* < .0001	*F*_(2,533)_ = 0.39, *p* = .675	Control vs. ED	0.97
	ED	124	21.90 (3.15)	23.30 (3.37)				ED vs. No ED	−0.39
	No ED	148	21.90 (3.01)	22.10 (3.09)				Control vs. No ED	0.56
Maladaptive coping (Brief‐COPE)	WCG	264	14.1 (3.11)	14.80 (3.18)	*F*_(2,341.47)_ = 1.46, *p* = .228	*F*_(2,299.03)_ = 6.33, *p* < .01	*F*_(2,233.72)_ = 0.88, *p* = .415	Control vs. ED	−0.56
	ED	124	14.70 (3.46)	13.60 (2.44)				ED vs. No ED	0.06
	No ED	148	14.10 (2.76)	13.80 (2.39)				Control vs. No ED	−0.53
Instrumental coping (Brief‐COPE)	WCG	264	9.85 (2.88)	9.58 (2.84)	*F*_(1,533)_ = 0.45, *p* = .501	*F*_(2,533)_ = 3.63, *p* < .05	*F*_(2,533)_ = 0.44, *p* = .646	Control vs. ED	0.27
	ED	124	9.40 (2.92)	9.91 (3.1)				ED vs. No ED	−0.26
	No ED	148	9.60 (2.85)	9.35 (2.82)				Control vs. No ED	0.01
Anxiety (GAD‐7)	WCG	265	10.19 (5.55)	10.00 (5.51)	*F*_(1,540)_ = 6.75, *p* < .01	*F*_(2,540)_ = 2.44, *p* = .089	*F*_(2,540)_ = 5.52, *p* < .01	Control vs. ED	−0.22
	ED	125	9.54 (5.62)	8.29 (5.15)				ED vs. No ED	0.17
	No ED	153	8.76 (5.24)	8.34 (5.13)				Control vs. No ED	−0.06
Depression (PHQ‐9)	WCG	242	11.00 (5.10)	11.70 (5.33)	*F*_(1,314.13)_ = 0.14, *p* = .705	*F*_(2,209.53)_ = 3.08, *p* < .05	*F*_(2,213.14)_ = 3.15, *p* < .05	Control vs. ED	−0.37
	ED	113	10.3 (5.42)	9.09 (4.69)				ED vs. No ED	0.27
	No ED	138	9.58 (4.68)	9.73 (3.89)				Control vs. No ED	−0.11
Global EC (PEC)	WCG	272	3.32 (0.28)	3.27 (0.25)	*F*_(1,285.67)_ = 12.97, *p* < .0001	*F*_(2,195.73)_ = 20.50, *p* < .0001	*F*_(2,227.24)_ = 1.11, *p* = .331	Control vs. ED	1.08
	ED	125	3.18 (0.25)	3.42 (0.28)				ED vs. No ED	−0.78
	No ED	152	3.32 (0.22)	3.38 (0.28)				Control vs. No ED	0.43
Intrapersonal EC	WCG	272	3.20 (0.36)	3.14 (0.29)	*F*_(1,301.32)_ = 17.92, *p* < .001	*F*_(2,205.90)_ = 8.80, *p* < .001	*F*_(2,219.84)_ = 1.77, *p* = .174	Control vs. ED	1.03
	ED	125	3.07 (0.35)	3.38 (0.34)				ED vs. No ED	−0.70
	No ED	152	3.22 (0.30)	3.30 (0.35)				Control vs. No ED	0.41
Interpersonal EC	WCG	272	3.44 (0.31)	3.40 (0.27)	*F*_(1,294.18)_ = 7.37, *p* < .01	*F*_(2,201.56)_ = 7.94, *p* < .001	*F*_(2,227.04)_ = 0.48, *p* = .620	Control vs. ED	0.67
	ED	125	3.30 (0.32)	3.47 (0.30)				ED vs. No ED	−0.34
	No ED	152	3.42 (0.27)	3.49 (0.29)				Control vs. No ED	0.37
Global trait EI (TEIQue‐SF)	WCG	134	4.50 (0.56)	4.52 (0.60)	*F*_(1,146.57)_ = 7.82, *p* < .01	*F*_(2,99.59)_ = 6.31, *p* < .01	*F*_(2,111.33)_ = 3.03, *p* = .053	Control vs. ED	0.68
	ED	60	4.21 (0.55)	4.55 (0.62)				ED vs. No ED	−0.65
	No ED	79	4.67 (0.51)	4.69 (0.57)				Control vs. No ED	−0.09
Emotionality	WCG	207	4.25 (0.59)	4.00 (0.53)	*F*_(1,231.06)_ = 23.97, *p* < .0001	*F*_(2,155.34)_ = 7.85, *p* < .001	*F*_(2,165.66)_ = 3.13, *p* < .05	Control vs. ED	1.31
	ED	84	3.93 (0.54)	4.44 (0.51)				ED vs. No ED	−0.66
	No ED	119	4.35 (0.52)	4.51 (0.51)				Control vs. No ED	0.72
Self‐control	WCG	210	3.83 (1.24)	3.94 (1.13)	*F*_(1,426)_ = 18.99, *p* < .0001	*F*_(2,426)_ = 4.78, *p* < .01	*F*_(2,426) =_ 2.57, *p* = .077	Control vs. ED	0.29
	ED	102	3.78 (1.28)	4.25 (1.28)				ED vs. No ED	−0.24
	No ED	117	4.09 (1.19)	4.25 (1.11)				Control vs. No ED	0.05
Sociability	WCG	207	4.87 (0.71)	4.82 (0.64)	*F*_(1,240.68)_ = 0.28, *p* = .597	*F*_(2,165.61)_ = 1.71, *p* = .184	*F*_(2,179.51)_ = 1.99, *p* = .139	Control vs. ED	0.35
	ED	99	4.54 (0.71)	4.74 (0.76)				ED vs. No ED	−0.38
	No ED	113	4.92 (0.67)	4.86 (0.59)				Control vs. No ED	−0.01
Well‐being	WCG	206	5.15 (0.83)	5.07 (0.97)	*F*_(1,269.55)_ = 0.04, *p* = .850	*F*_(2,181.91)_ = 3.59, *p* < .05	*F*_(2,186.22)_ = 2.64, *p* = .074	Control vs. ED	0.27
	ED	103	4.68 (0.91)	4.90 (0.96)				ED vs. No ED	−0.26
	No ED	117	5.26 (0.79)	5.09 (0.72)				Control vs. No ED	0.01
Well‐being (WHO‐5)	WCG	264	9.47 (3.49)	9.48 (3.82)	*F*_(1,298.99)_ = 8.05, *p* < .01	*F*_(2,207.82)_ = 2.91, *p* = .057	*F*_(2,207.83)_ = 1.26, *p* = .285	Control vs. ED	0.43
	ED	125	9.48 (3.51)	11.00 (3.48)				ED vs. No ED	−0.20
	No ED	153	9.75 (4.1)	10.50 (3.95)				Control vs. No ED	0.20

Abbreviations: *d* > 0.10, small effect size; *d* > 0.50, moderate effect size; *d* > 0.80, large effect size (Cohen, 1988); EC, emotional competencies; ED, intervention group with emotional diary; no ED, intervention group without ED; WCG, waitlist comparison group.

**FIGURE 1:**
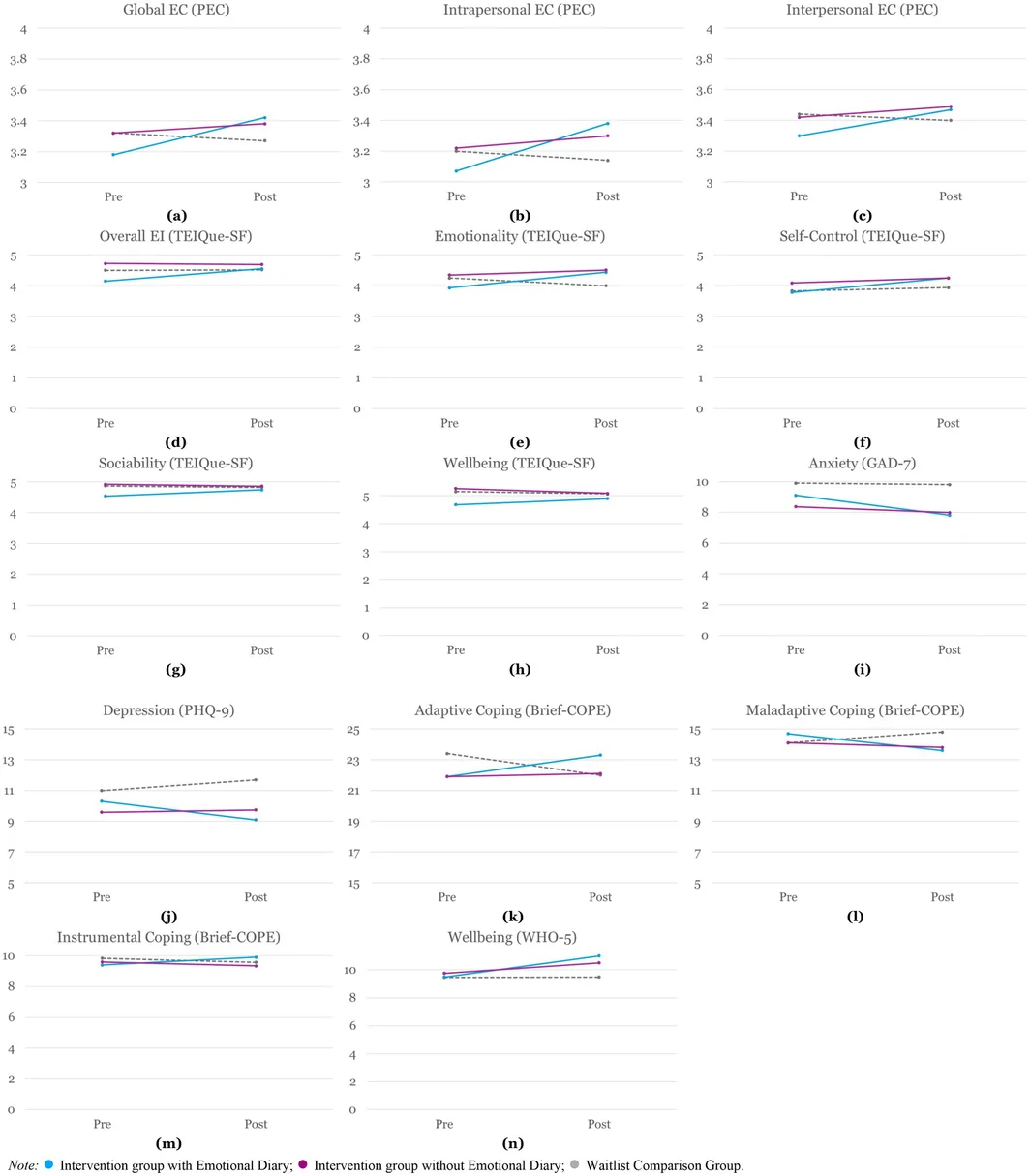
Pre–post intervention changes across all outcome variables by study group (waitlist comparison, YEY without ED, and YEY with ED). (a) Pre–post intervention changes across global EC (PEC). (b) Pre–post intervention changes across intrapersonal EC (PEC). (c) Pre–post intervention changes across interpersonal EC (PEC). (d) Pre–post intervention changes across overall EI (TEIQue‐SF). (e) Pre–post intervention changes across emotionality (TEIQue‐SF). (f) Pre–post intervention changes across self‐control (TEIQue‐SF). (g) Pre–post intervention changes across sociability (TEIQue‐SF). (h) Pre–post intervention changes across well‐being (TEIQue‐SF). (i) Pre–post intervention changes across anxiety (GAD‐7). (j) Pre–post intervention changes across depression (PHQ‐9). (k) Pre–post intervention changes across adaptive coping (Brief‐COPE). (l) Pre–post intervention changes across maladaptive coping (Brief‐COPE). (m) Pre–post intervention changes across instrumental coping (Brief‐COPE). (n) Pre–post intervention changes across well‐being (WHO‐5). ● Intervention group with emotional diary; ● Intervention group without emotional diary; ● Waitlist comparison group.

Similar trends were found for Intrapersonal emotional competence (PEC; Figure 1b), with a significant interaction (*F*_(2,205.90)_ = 8.80, *p* < .001), and effect sizes indicating a large effect for ED versus comparison (*d* = 1.03), small for non‐ED versus comparison (*d* = 0.41) and moderate between intervention groups (*d* = −0.70). For interpersonal emotional competence (PEC; Figure 1c), the waitlist comparison group remained stable, while both intervention groups improved, especially the ED group. The interaction was significant (*F*_(2,201.56)_ = 7.94, *p* < .001), with moderate effect sizes for ED versus comparison (*d* = 0.67), small for non‐ED versus comparison (*d* = 0.37) and a small effect between the intervention groups (*d* = −0.37).

Overall TEIQue scores (Figure 1d) increased in the ED group, remained stable in the waitlist comparison group and slightly decreased in the non‐ED group. A significant interaction was observed (*F*(2,99.59) = 6.31, *p* < .01), with moderate effect sizes for ED versus comparison (*d* = 0.68), trivial for non‐ED versus waitlist comparison group (*d* = −0.09) and large between intervention groups (*d* = −0.81).

Among TEIQue factors, interaction effects were significant for emotionality (Figure 1e), self‐control (Figure 1f) and well‐being (Figure 1h), but not for sociability (Figure 1g). Emotionality showed a large effect in favour of ED (*d* = −1.31) and moderate differences for the other comparisons. This finding should nevertheless be interpreted cautiously, given the relatively low internal consistency of the emotionality subscale in the present sample, although low reliability would typically be expected to attenuate observed effects. Other subscales showed small effect sizes favouring ED versus comparison (range *d* = 0.27–0.35), with negligible or small differences between the intervention groups.

#### Mental health‐related variables

Wellbeing scores (Figure 1n) remained stable in the waitlist comparison group and increased in both intervention groups, especially in the ED group. Although the interaction was marginally non‐significant (*F*_(2,207.82)_ = 2.91, *p* = .057), effect sizes showed small effects for ED versus comparison (*d* = 0.43) and non‐ED versus comparison (*d* = 0.20).

Anxiety scores (Figure 1i) remained stable in the waitlist comparison group and decreased in both intervention groups, more so in the ED group. Although the interaction effect was not significant (*F*_(2,540)_ = 2.44, *p* = .089), main effects were found for time (*F*_(1,540)_ = 6.75, *p* < .01) and group comparisons (*F*_(2,540)_ = 5.52, *p* < .01). Effect sizes indicated a small reduction in anxiety for ED versus comparison (*d* = −0.22), a negligible effect for non‐ED versus waitlist comparison group (*d* = −0.06) and a very small effect favouring ED for ED versus non‐ED (*d* = 0.17).

For depression symptoms (PHQ‐9; Figure 1j), the waitlist comparison group showed a slight increase, while the non‐ED intervention group remained relatively stable. In contrast, the ED intervention group exhibited a decreasing trend. The ANOVA interaction effect was significant (*F*_(2,209.53)_ = 3.08, *p* < .05), with a small‐to‐moderate effect size for ED versus waitlist comparison group (*d* = −0.37).

#### Coping variables

Adaptive coping (Figure 1k) decreased in the waitlist comparison group, remained stable in the non‐ED group and increased in the ED group. The interaction was significant (*F*_(2,533)_ = 16.26, *p* < .001), with moderate‐to‐large effects favouring both intervention groups versus waitlist comparison group (ED: *d* = 0.97; non‐ED: *d* = 0.56) and a small effect between intervention groups (*d* = −0.39). Maladaptive coping (Figure 1l) increased in the waitlist comparison group and decreased in both intervention groups. A significant interaction was found (*F*_(2,299.03)_ = 6.33, *p* < .01). Effect sizes were moderate for ED versus waitlist comparison group (*d* = −0.56) and non‐ED versus waitlist comparison group (*d* = −0.53), with a negligible difference between intervention groups (*d* = 0.06).

Instrumental coping (Figure 1m) remained stable in the waitlist comparison group and non‐ED groups and increased in the ED group. The interaction was significant (*F*_(2,533)_ = 3.63, *p* < .05), with small effects favouring the ED group compared to the others (ED vs. waitlist comparison group: *d* = 0.27; ED vs. non‐ED: *d* = 0.01).

## STUDY 2: QUALITATIVE PHASE

### Study 2: Method

#### Data collection

The qualitative component focused on adolescents' emotional development within a context of sociopolitical adversity, complementing previous diary‐based research on emotion regulation in everyday life. Qualitative data were derived from emotional diary (ED) entries and analysed using codebook thematic analysis (Braun & Clarke, 2006), an approach well suited to explanatory mixed‐methods research because it combines systematic coding with theoretically informed interpretation.

##### Emotional diaries

As part of the qualitative component of the study, participants receiving the intervention completed an ED after each session. Participants who completed the EDs and submitted them during the following sessions were included in the intervention group with ED. Conceived as both an evaluative and educational tool, the ED was aligned with the objectives of the YEY programme and the PEC Model (Brasseur et al., 2013). It was designed to foster self‐reflection and emotional articulation across five domains of emotional competence through six open‐ended questions: (1) *What happened?*—to describe a specific event and contextualize emotional and behavioural responses; (2) *What emotions did you feel?*—to identify and label emotions, reinforcing emotion recognition and regulation; (3) *What were your thoughts?*—to promote self‐reflection (Ullrich & Lutgendorf, 2002); (4) *What did you do?*—to describe behavioural responses and coping strategies (Compas et al., 2017); (5) *What were the consequences? and What did you learn?*—to encourage processing, learning and well‐being (Moffitt et al., 2023); and (6) *tell us if you applied anything learned from the sessions, and if so, how?*—to enhance emotional insight and growth. These questions were developed according to the research interests and objectives of the study, grounded in the experience, literature review and consensus of the research team, and inspired by previous studies employing ED as pedagogical and psychological tools (e.g., Hanson, 2019; Rossman & Rallis, 2011).

The diary was designed to guide adolescents step by step through emotional reflection, and its cultural relevance was reinforced by allowing references to familiar, everyday situations (e.g., family meals, school exams or local events). Instructions emphasized honesty, openness and a non‐judgmental attitude, promoting a safe space for introspection. Overall, the ED provided a metacognitive space for participants to consolidate emotional learning, practice emotional competencies and increase awareness of internal processes, while simultaneously serving as a source of qualitative data for the study.

#### Data analysis

The diaries were originally written in Arabic and translated into English prior to analysis by bilingual members of the research team with proficiency in both languages. This process aimed to preserve the semantic meaning and contextual nuances of participants' narratives while ensuring consistency throughout the analysis.

Qualitative data were analysed using a codebook thematic analysis, drawing on the six‐phase framework proposed by Braun and Clarke (2006). Given the explanatory purpose of the qualitative component within the mixed‐methods design, this approach enables the systematic organization of participants' accounts while remaining sufficiently flexible to identify meaningful patterns across the data set. Unlike reflexive thematic analysis, which prioritizes the interpretative generation of themes, the codebook approach combines systematic coding procedures with theoretically informed interpretation, making it particularly suitable for studies in which qualitative findings are intended to complement and contextualize quantitative results. The analysis was guided by the following research question: *How does engagement with emotional diary writing support emotional competencies within the intervention, and how can its contribution be understood in relation to the programme's quantitative outcomes?* Consequently, the qualitative analysis sought to identify recurring patterns in participants' narratives that could help explain the changes observed in the quantitative measures following participation in the intervention.

During the initial stages of the analysis, both researchers repeatedly read the diaries to familiarize themselves with the data and generated initial codes directly from participants' narratives without imposing predefined categories. Subsequently, these codes were organized into broader analytic domains through an iterative process informed by both the research objectives and the emotional competence framework (Brasseur et al., 2013). Thus, while coding remained grounded in participants' accounts, the organization of the final analytic domains was theoretically informed and aligned with the explanatory aims of the study.

Data management and coding were supported using Atlas.ti software. To enhance methodological transparency and consistency in the application of the coding framework, two researchers independently coded a subset of the diary entries. Before formal coding, both researchers jointly reviewed the preliminary coding framework and discussed code definitions to ensure a shared understanding of the analytic procedures. Coding discrepancies were subsequently discussed until consensus was reached, leading to the refinement of the coding framework where necessary. The remaining data were then coded using the agreed codebook, while regular meetings were held throughout the analytic process to discuss ambiguous cases and ensure consistent application of the coding framework. Cohen's kappa indicated high agreement (Cohen's *κ* = .812, SE = .053, *p* < .001; *N* = 992 coded units), suggesting that the coding framework was applied consistently across researchers, rather than as evidence of a single objective interpretation of the data.

Throughout the analysis, an audit trail was maintained documenting coding decisions, revisions to the codebook and the development of the analytic domains. Regular discussions among the research team encouraged critical reflection on coding decisions, interpretation of participants' narratives and the integration of the qualitative findings with the quantitative results. Importantly, the researchers responsible for the qualitative analysis were not involved in delivering the intervention, reducing the possibility that expectations regarding programme effectiveness influenced the interpretation of participants' accounts. The final analysis identified five overarching analytic domains that captured recurrent patterns across participants' emotional narratives and provided contextual insight into the quantitative findings concerning emotional competence, psychological well‐being and coping during participation in the YEY programme.

### Study 2: Findings

The codebook thematic analysis identified five overarching analytic domains that captured recurrent patterns in participants' emotional narratives and provided contextual understanding of the quantitative findings. These domains comprised intrapersonal emotional competence, interpersonal emotional competence, anxiety, depressive symptoms and adaptive/maladaptive coping.

#### Analytic domain 1: Intrapersonal emotional competence

In relation to intrapersonal emotional competence, participants' accounts in the initial sessions frequently reflected difficulties in emotional processes, particularly in emotional regulation (*‘I was very mad; my body was agitated […] I learned not to express my emotions.’*, MS24), a lack of optimism when facing life situations that could be subjectively considered difficult (‘*Stress, frustration, fear of failing, not succeeding in the future‐ I cried*,’ LM08), or manifestations reflecting diminished self‐esteem with a lack of self‐confidence that ultimately significantly harms the person's well‐being, as in the case of RA01 (‘*I froze and I felt shy at first and after a few seconds […] I don't have enough confidence in myself and that I'm weak*.’).

As the sessions progressed, some participants described changes in how they understood and managed their emotions. For example, RA01 described a shift in self‐perception following positive family experiences (*‘I felt important. I felt as if I were an efficient member of the family’*). Similarly, participant AA23 reported increased self‐awareness and decision‐making regarding interpersonal relationships (*‘I thought I deserved a better person so […] stay away from that person and think less about them. I used this paper to express myself and investigate and talk to myself in front of a mirror’*). AN30 reported attempts to regulate intense emotions, although these were not always described as fully effective (*‘I cried a lot and yelled. [I learned] to try not to get nervous’*). Participant CW04 reported increased awareness of emotional states, particularly in identifying causes of sadness or demotivation (*‘Super sad, demotivated, mentally tired and physically, constantly crying then slept. […] I now know how to identify what and why I'm feeling this particular way’*), while also indicating ongoing challenges.

In addition to emotional regulation and self‐esteem, some participants reported increased motivation or perseverance over time. For instance, ZZ15 stated (‘*I felt happy and encouraged. I did it and I will do it again*.’). These accounts suggest that, for some participants, engagement with the diary was associated with changes in self‐perception and emotional awareness, although this pattern was not uniform across all cases.

#### Analytic domain 2: Interpersonal emotional competence

Compared to intrapersonal emotional competence, changes in interpersonal emotional competence were less frequently reported, although some participants described developments in recognizing others' emotions, empathy and managing interpersonal conflicts. As the intervention progressed, some accounts reflected changes in how participants responded to social situations. For example, AR26 initially described difficulty understanding others' emotions and responded with withdrawal (‘*I felt a little jealous and upset […] and ignored them*’), while later describing a situation involving acknowledgement of personal responsibility (‘*I got upset and ignored them – I apologized*’). These accounts may reflect increased attention to interpersonal dynamics and emotional communication, although such changes were not consistently observed across all participants.

#### Analytic domain 3: Anxiety

Anxiety was frequently reported in the initial sessions, often in relation to academic pressure, fear of failure and interpersonal concerns. Narratives such as LM08 (‘*Stress, frustration, fear of failing, not succeeding in the future*’) and CH26 (‘*Anxious, scared, and a little bit angry because I did not pay attention to a lot of easy questions*’) illustrate high levels of emotional distress and limited reported strategies for managing these experiences at baseline.

In later sessions, some participants described using strategies aimed at managing these emotional states. For example, HT26 initially reported stress and early attempts at regulation (‘*I was worried and stressed […]. I did things that made me feel relaxed*’) and later described the use of specific techniques: ‘*I used the breathing technique. […] I feel proud and happy. I felt my body in good health and very comfortable*.’ Similarly, LM08 reported: ‘*I tried the breathing method when I'm worried and tried the activity to close bad thoughts I am having*.’

#### Analytic domain 4: Depressive symptoms

Accounts from the initial sessions included expressions of sadness and negative self‐evaluation, particularly following academic or interpersonal difficulties. For example, LD22 stated (‘*I felt depressed, sad, and stupid*’), and CW04 described (‘*Overwhelmed. Want to give up. Crying. Sleeping*.’). These accounts reflect the presence of distressing emotional experiences at the beginning of the intervention.

In subsequent sessions, some participants described changes in how they responded to these experiences, including more constructive or future‐oriented perspectives. For instance, LD22 stated (‘*After that I decided not to quit and study more*’). While such accounts suggest shifts in how some participants interpreted their experiences, these changes were not observed consistently across all cases.

#### Analytic domain 5: Adaptive/maladaptive coping

In the initial phase (Sessions 1–4), participants frequently reported coping responses that could be characterized as maladaptive, including venting (LM08: ‘*Stress, frustration, fear of failing, not succeeding in the future‐ I cried’*), behavioural disengagement (CW04: *‘Overwhelmed. Want to give up. Crying. Sleeping’*) and self‐blame (CH26: ‘*Anxious, scared, and a little bit angry because I did not pay attention to a lot of easy questions*’), related to negative emotions such as sadness, anxiety and frustration.

As sessions progressed, some participants described a shift towards the use of coping strategies that may be considered more adaptive. Between sessions 5 and 7, participants reported strategies such as active coping (AN30: ‘*I was disappointed, nervous, and scared. I Cried a lot and yelled. (I learned) to study well for the exam, to review, and to try not to get nervous’*), positive reinterpretation (LM08: *‘I felt mentally and physically tired and I learned that everything needs patience’*) and seeking emotional support (CH26: *‘I tried to calm down and my friend helped me’*). In later sessions, some accounts reflected continued use of these strategies, as illustrated by CW04: ‘*Getting low grades […] Sad, tired, frustrated, devastated. I tried, grades do not define me, scared for uni. Slept and went out. It happens; I'll try harder for next time’*—reflecting coping strategies such as acceptance and positive reinterpretation.

#### Changes in emotional experiences and coping strategies

Table 3 was developed after completion of the coding process by reviewing participants' diary entries across the three phases of the intervention. Rather than quantifying the occurrence of codes, it provides a descriptive longitudinal synthesis illustrating how the recurrent analytic domains identified during the Codebook Thematic Analysis were reflected in participants' narratives over time (Sessions 1–4, Sessions 5–7 and Sessions 8–11). The table provides a descriptive synthesis of the predominant pattern identified in each phase of each participant's diary entries. Colour coding was used as a visual aid to represent low adjustment/distress, mixed or transitional patterns and improved adjustment over time. In the initial phase, many participants reported experiences consistent with anxiety, depressive feelings, maladaptive coping strategies and difficulties in emotional regulation, reflecting the types of challenges described at baseline. Between Sessions 5 and 7, some participants began to report changes in how they described and managed their emotional experiences. For example, participants such as AN30, AR26 and CH26 reported a shift from earlier difficulties towards references to more adaptive coping strategies and greater emotional awareness. Similarly, CW04 and HT26 described experiences suggesting increased attention to emotional regulation and more positive interpretations of situations, although these patterns were not uniform across all cases.

**TABLE 3:** Descriptive synthesis of reported emotional experiences across intervention phases (S1–S11).

	S. 1≈4	S. 5≈7	S. 8≈11	Observations
AA21		●	●	From anxiety → to the use of adaptive coping, improved self‐control and increased self‐motivation.
AN30	●	●	●	From maladaptive coping and poor emotional control/expression → to the use of adaptive coping strategies and better self‐regulation.
AR26		●	●	From anxiety, maladaptive coping and dysregulation → to the use of adaptive coping, and greater optimism and self‐motivation
BR13		●	●	No signs of psychological or emotional improvement were observed.
CH26	●	●		From maladaptive coping and anxiety → to the use of adaptive coping, improved emotion regulation and reduction of anxiety symptoms.
CW04		●	●	From anxiety, depressive symptoms and maladaptive coping → to the use of adaptive coping and increased self‐motivation.
DB03		●	●	From depressive symptoms → to increase emotional awareness and understanding, enhanced self‐regulation, empathy and use of adaptive coping.
HT26		●	●	From anxiety, depressive symptoms and poor self‐control → to better well‐being, optimism, happiness and self‐motivation.
IM16	●	●	●	From anxiety, depressive symptoms and maladaptive coping → greater emotional awareness and support‐seeking, although some maladaptive coping remained.
LD22		●	●	From depressive symptoms and maladaptive coping → to improved well‐being, higher self‐esteem and increased regulation and adaptive coping
LM08	●	●	●	From anxiety, depressive symptoms and poor self‐control→ to greater acceptance, positive reframing, active coping and better regulation, although some anxiety and depressive symptoms persisted.
MA27	●	●	●	From depressive symptoms, maladaptive coping, low self‐esteem and poor emotional regulation → to the use of active coping strategies.
MS24	●	●	●	From anxiety, depressive symptoms and maladaptive emotional expression → to the use of adaptive coping and more appropriate emotional expression.
SH28		●	●	From anxiety, depressive symptoms and maladaptive coping → to adoption of adaptive coping and greater self‐motivation.
TA27	●	●	●	From poor emotional expression and regulation → to greater awareness and regulation efforts, but emotional dysregulation remained.
ZZ15	●	●	●	From maladaptive coping, depressive symptoms and poor self‐control → to enhanced well‐being, self‐motivation and self‐esteem.

*Note*: The colour coding (red, amber, green) reflects the presence of indicators of emotional distress, mixed adjustment or adaptive functioning within each phase, based on qualitative coding of diary entries. ● Low adjustment/indicators of psychological distress or maladaptive coping; ● Moderate adjustment/mixed signs or partial transition; ● High adjustment/clear indicators of emotional regulation, active coping, well‐being or improvement. Observations summarize participants' reported experiences and should be interpreted as illustrative rather than causal or generalizable patterns.

By Sessions 8–11, some participants reported more consistent use of coping strategies and greater emotional awareness in their entries. For instance, LM08, MA27 and ZZ15 included references to active coping strategies and reflections related to self‐perception, while participants such as TA27 continued to report challenges alongside attempts to regulate and express emotions more effectively. Overall, the data suggest a shift in some cases from earlier reports of distress or maladaptive coping towards later references to more adaptive strategies, although this pattern varied across participants.

The table also highlights considerable variability in participants' reported experiences. While some individuals (e.g., AN30, CW04) described more consistent changes across phases, others (e.g., BR13) did not report clear changes in their emotional experiences over time. These differences indicate that participants engaged with and described their experiences of the intervention in diverse ways and should be interpreted with caution.

## DISCUSSION

At‐risk youth are in need of psychosocial interventions that reduce the impact of stressors and improve overall mental health and functioning. Among the domains that are most important for adolescents' development is trait EI. Here, we provide empirical evidence, both quantitative and qualitative, that supports the efficacy of the YEY programme as a socio‐emotional intervention among adolescents living in vulnerable conditions in Lebanon. By integrating an emotional diary (ED) within a mixed‐methods design, the study also explored its potential contribution as both a pedagogical and reflective tool. Overall, the findings indicate positive patterns of change in trait EI, adaptive coping and well‐being. The findings highlight both the relevance and protective potential of culturally contextualized SEL initiatives in non‐Western, low‐resource contexts.

### General contributions and socio‐emotional development

The findings provide evidence that the YEY programme is a meaningful and feasible socio‐emotional intervention for Lebanese adolescents. Significant gains were observed in EI, coping and well‐being, especially when the ED component was included (Armstrong‐Carter et al., 2020). The inclusion of a waitlist comparison group strengthens the interpretation of these patterns at the programme level.

With respect to the ED component, participants who engaged in the diary tended to show more consistent patterns of improvement across several outcomes, particularly when compared to the waitlist comparison group. However, given that participation in the ED was voluntary, these differences should be interpreted with caution, as they may reflect not only the influence of the diary itself but also differences in motivation, engagement or baseline characteristics among participants. Although some pre‐intervention differences were observed between the diary and non‐diary intervention groups, these were limited to a small number of variables and were small in magnitude, and effect sizes for those outcomes were estimated using an adjusted procedure that accounts for baseline differences. Moreover, the more pronounced improvements in the diary condition relative to the non‐diary intervention group were observed across key quantitative outcomes (e.g., global trait EI and global and intrapersonal EC), but not all of them, and should therefore be considered outcome‐specific rather than general. Rather than establishing any causal effect, the findings suggest that the ED may function as a supportive tool within the intervention, facilitating emotional reflection and engagement. This is consistent with prior literature indicating that reflective practices can contribute to socio‐emotional development, although their effects are often context‐dependent and influenced by individual differences (Harding et al., 2019).

Overall, the observed improvements in intrapersonal and interpersonal emotional competence, enhanced adaptive coping and reductions in depressive symptoms may buffer adolescents in chronically adverse contexts. From a developmental perspective, these gains during adolescence—a period of heightened neuroemotional plasticity—hold potential to shape long‐term trajectories of adaptation and resilience (Chowdhury et al., 2023; Soto & Tackett, 2015).

### Trait EI and socio‐emotional competencies

From a quantitative perspective, adolescents in the intervention group with ED showed improvements in trait EI (measured by both TEIQue and PEC), with larger effect sizes relative to the waitlist comparison group. These effects were also, in some cases, greater than those observed in the intervention group without ED. While these patterns are consistent with theoretical accounts that highlight the positive role of emotional writing in adolescent development (Bermejo‐Martins et al., 2018; Mortari et al., 2024; Utari et al., 2023), they cannot be interpreted as evidence of a direct causal effect of the ED, given the self‐selection into this condition. Improvements were particularly evident in intrapersonal domains such as emotional regulation and global trait EI, which may reflect the role of reflective activities in promoting emotional awareness. Gains in interpersonal domains were more modest but still suggest developments in empathy and emotional understanding in the ED group.

The qualitative findings provided contextual evidence that helped explain the quantitative improvements observed in emotional competence, coping and psychological well‐being. However, as these findings are based on self‐reported narratives, they should be interpreted as context‐dependent and illustrative rather than as objective indicators of change. In addition, although intervention conditions were similar across groups except for the journaling component, the non‐random allocation of participants to the ED condition limits the extent to which observed differences can be attributed solely to the diary.

### Coping strategies and psychological well‐being

Regarding mental health outcomes, both intervention conditions exhibited modest reductions in anxiety and depressive symptoms relative to the waitlist comparison group. The ED group showed more consistent patterns of improvement in some outcomes, including well‐being, although these differences were not always statistically significant. While anxiety reductions did not reach statistical significance, their direction and consistency suggest potential clinical relevance. The pattern of improvement supports the buffering role of emotional reflection in mitigating distress (Guo, 2023; Sohal et al., 2022). These patterns may suggest that reflective practices such as journaling can support emotional processing and coping.

Coping patterns also showed changes over time. Participants in the ED group reported increased use of adaptive coping strategies and reduced reliance on maladaptive strategies. These findings are consistent with previous literature on reflective writing and coping (Tonarelli et al., 2017). Although the group without ED also improved slightly, the added benefit of journaling underscores its value in reinforcing emotional learning and flexibility (Kotsou et al., 2019). Qualitative evidence helped explain this interpretation: adolescents progressively moved from venting and self‐blame towards active coping, help‐seeking and positive reinterpretation. Again, these accounts reflect reported experiences and should be interpreted within the context of the study design. Taken together, findings suggest that integrating reflective practices such as ED within SEL programmes may offer added value in supporting adolescents' emotional awareness and coping. However, further research using designs that allow for stronger causal inference (e.g., randomized allocation to diary conditions) would be necessary to more clearly establish the specific contribution of journaling. In contexts characterized by adversity and limited resources, even modest improvements may be meaningful, particularly when interventions are feasible and culturally adapted.

### Research and practical implications

This study provides novel evidence of the effectiveness of a culturally adapted, SEL‐based intervention implemented in a high‐adversity context. By combining quantitative and qualitative methods, the findings suggest that structured emotional programmes may support adolescents' emotional competencies, coping strategies and overall well‐being in vulnerable contexts. The inclusion of reflective tools such as the emotional diary (ED) was associated with more consistent patterns of improvement across some outcomes, although these patterns should be interpreted with caution given the voluntary nature of participation.

Practically, the findings indicate the potential value of incorporating ED into SEL initiatives. This low‐cost and easily scalable tool deepens emotional awareness and regulation, making it especially relevant for educators and youth practitioners operating in resource‐limited environments. However, differences observed between participants who engaged with the diary and those who did not may also reflect variations in motivation, engagement and other baseline characteristics and therefore should not be interpreted as evidence of a direct causal effect. The participatory, youth‐led design of the programme also appeared to play an important role in supporting engagement and involvement, suggesting that implementation features may be as relevant as specific components.

From a methodological standpoint, the study employed robust and contemporary statistical techniques, including trimmed means and a corrected version of Cohen's *d* for repeated measures. This analytical approach enhanced the precision and interpretability of the results, allowing for a more nuanced understanding of patterns of change across groups while acknowledging the limitations inherent to the study design.

### Limitations and recommendations for future research

Despite its promising outcomes, several limitations must be acknowledged. First, the intervention was conducted within a specific cultural and educational context to address an underrepresented population experiencing significant sociopolitical and economic challenges, although this may limit the generalizability of the findings to other populations. Future research would therefore benefit from cross‐cultural replications to examine the applicability of these findings across diverse contexts. In addition, the sample spanned a relatively broad developmental period (mid‐adolescence to emerging adulthood), which may involve psychological differences. Although baseline differences in age and gender were observed across groups, no consistent moderating effects of these variables were found, suggesting that intervention effects were broadly robust across these demographic characteristics. Nevertheless, future studies should consider more developmentally homogeneous samples and longitudinal designs to better capture developmental changes.

Methodologically, the quantitative data relied primarily on self‐report measures, which may have introduced mono‐method bias, as well as social desirability. Furthermore, the absence of long‐term follow‐up data in the present study limits conclusions regarding the sustainability of the observed benefits over time. In addition, the quasi‐experimental design and group allocation procedures limit causal interpretation. Schools, rather than individuals, were assigned to conditions, and participation in the emotional diary (ED) component was voluntary. Although baseline comparisons indicated that the ED and non‐ED groups were broadly similar across most variables, and the few significant pre‐intervention differences observed were small in magnitude, participants who engaged in the ED may still have differed in unmeasured characteristics such as motivation, emotional engagement or willingness to reflect on personal experiences. Therefore, differences observed between the ED and non‐ED groups should be interpreted cautiously and cannot be attributed solely to the diary component.

Finally, although the global trait EI score showed good reliability, the lower internal consistency observed for some of the TEIQue factors—particularly emotionality and, to a lesser extent, sociability—suggests that findings at the subscale level should be interpreted with caution. Accordingly, the results are more robust for the global trait EI score and for the broader PEC indices than for the less reliable TEIQue subdimensions. In addition, factor‐ and facet‐level analyses of trait EI are ideally conducted using the full form of the TEIQue (Cooper & Petrides, 2010). At the same time, it is worth noting that lower reliability would generally be expected to attenuate rather than inflate observed effects, so the significant emotionality finding should not be dismissed outright but interpreted cautiously as exploratory.

Future research should aim to replicate these findings across diverse cultural and socioeconomic contexts and to assess the long‐term outcomes of SEL programmes incorporating reflective components such as ED. In particular, designs allowing for random assignment to diary conditions would help establish the specific contribution of journaling more clearly. Exploring the moderating and mediating role of other individual factors, such as exposure to adversity or character strengths, may further clarify for whom and under what conditions these interventions are most beneficial. Finally, including additional informants (e.g., teachers, parents) and complementary behavioural or physiological indicators could provide a more comprehensive understanding of emotional development processes.

## CONCLUSIONS

This study provides evidence of the effectiveness of the *Yes to Emotions (YEY)* programme in enhancing trait EI, coping strategies and psychological well‐being among adolescents facing social vulnerability. Through a mixed‐methods design, the intervention demonstrated measurable improvements in both self‐reported emotional competencies and participants' subjective emotional narratives—particularly when the programme included a structured ED component. These findings highlight not only the overall value of SEL in adolescent development but also the potential added value of reflective practices in fostering emotional insight, emotion regulation and resilience.

The results are especially relevant in high‐stress, low‐resource settings like Lebanon, where adolescents often face cumulative stressors and limited access to psychosocial support. By empowering young people to recognize, express and manage their emotions, the YEY programme contributes to strengthening internal protective factors that can buffer the effects of chronic adversity. Moreover, the qualitative data revealed meaningful shifts in participants' emotional language, interpersonal awareness and self‐concept—suggesting that participants described personal changes in how they understood and managed their emotional experiences.

Beyond confirming the feasibility of implementing SEL‐based interventions in complex sociopolitical contexts, this study underscores the importance of integrating culturally responsive, youth‐centred and participatory approaches. The ED tool, in particular, emerged as a low‐cost and scalable strategy to promote deeper engagement and sustained emotional reflection. As global efforts to promote adolescent mental health and emotional literacy continue to grow, programmes like YEY offer promising and adaptable models for supporting youth well‐being in diverse and often challenging environments.

The integration of quantitative and qualitative findings strengthened the interpretation of programme effectiveness by combining evidence of measurable change with participants' subjective accounts of their emotional experiences. Whereas the quantitative analyses demonstrated improvements in emotional competence and coping, the qualitative findings provided contextual insight into how adolescents experienced these changes throughout the intervention.

In conclusion, this study provides evidence for the effectiveness of the YEY emotional intervention in promoting emotional competencies and adaptive coping among adolescents living in conditions of social vulnerability. The integration of emotional diaries was associated with more consistent improvements across several quantitative outcomes and provided valuable contextual insight into adolescents' emotional experiences throughout the intervention.

## AUTHOR CONTRIBUTIONS

**J. M. Gimenez‐Lozano:** Methodology; writing – review and editing; formal analysis; data curation. **A. Hernández‐Saguar:** Methodology; writing – review and editing; formal analysis; software; data curation. **K. Petrides:** Writing – review and editing; supervision; methodology. **M. Checa‐Romero:** Writing – original draft; writing – review and editing; validation; methodology; formal analysis; supervision. **M. J. Sanchez‐Ruiz:** Conceptualization; funding acquisition; writing – original draft; methodology; writing – review and editing; project administration; resources. **M. El Khoury‐Malhame:** Conceptualization; investigation; funding acquisition; writing – original draft; software; visualization. **S. El‐Hajj:** Conceptualization; investigation; funding acquisition; writing – original draft; resources. **R. Doumit:** Conceptualization; investigation; funding acquisition; writing – original draft; software; visualization.

## FUNDING INFORMATION

This research was funded by Grand Challenges Canada—Global Mental Health (ref. R‐GMH‐POC‐2107‐47498)—project title ‘Yes to Emotions in Youth (YEY): An Emotional Intelligence Training for Vulnerable Youth in Lebanon’. Maria‐Jose Sanchez‐Ruiz's work was supported by Ramón y Cajal program (RYC2020‐030471‐I) funded by the Spanish Ministry of Science and Innovation (MCIN/AEI/10.13039/501100011039) and FSE (Invierte en tu futuro).

## CONFLICT OF INTEREST STATEMENT

The authors declare no conflicts of interest.

## ETHICS STATEMENT

Ethical approval for this study was obtained from the Lebanese American University Institutional Review Board (LAU IRB; Ref: LAU.SAS.MJ1.28/Oct/2022). Administrative authorization for data collection was granted by the Lebanese Ministry of Education and Higher Education (MEHE). All procedures were conducted in accordance with the ethical standards of the institutional research committee and with the 1964 Helsinki Declaration and its later amendments.

## CONSENT

Written informed consent was obtained from the participants and parents of participating adolescents prior to data collection. Participation was voluntary, and all participants were informed of their right to withdraw at any point without any negative consequences.

## Supporting information


Table S1.

## Data Availability

Due to ethical and legal constraints related to research with minors, the data sets generated and/or analysed during the current study are not publicly available. De‐identified data are available from the corresponding author upon request.
