# Supplementary material for: The effectiveness of an emotional intelligence intervention on Lebanese adolescent mental health: A mixed‐method study with emotional diary integration
Source: Br J Dev Psychol. 2026 Jul 31;44(4):978–1003. doi: 10.1111/bjdp.70061 (PMC13648305; doi:10.1111/bjdp.70061)
Supplement: Supplementary file 1 [file BJDP-44-978-s001.docx]

**Supplementary Material**

**Supplementary Baseline Comparisons and Moderation Analyses**

Baseline comparisons were conducted across all groups, including both intervention groups and the waitlist comparison group. However, particular attention was given to comparisons between the two intervention groups, as participation in the emotional diary activity was self-selected rather than randomly assigned. Therefore, it was necessary to examine whether these groups were comparable at baseline.

Most pre-test differences between the two intervention groups were non-significant. Significant differences were found only for global emotional competence (*p* = .036, *d* = -0.32), interpersonal emotional competence (*p* = .044, *d* = -0.24), global trait EI (*p* = .006, *d* = -0.53), and TEIQue wellbeing (*p* = .011, *d* = -0.42). The magnitude of these differences was small for most variables and moderate for global trait EI, indicating limited but non-negligible baseline differences between the two intervention groups.

For this reason, intervention effect sizes were estimated using Morris’s (2008) adjusted *d*, which accounts for pretest scores when quantifying differential change over time.

Because baseline differences in age and gender distribution were observed across the three study groups, exploratory moderation analyses were conducted to examine whether these demographic variables influenced intervention effects over time. Specifically, moderation was tested through the interaction between change over time, study group, and each demographic variable.

As shown in Table S1, no consistent evidence of moderation was found for either age or gender. Age significantly moderated intervention effects only for intrapersonal emotional competence, global emotional competence, and Self-Control. However, these effects were small in magnitude (η_p_² = .017–.021) and were not consistently observed across related constructs. No significant moderation effects were observed for gender across any outcome. Overall, these results suggest that the main pattern of intervention effects was broadly robust across age and gender.

**Table S1.**

*Baseline comparisons and exploratory moderation analyses for age and gender.*

| **Outcome** | **Age moderation** | **Gender moderation** | **Baseline Differences** |
| --- | --- | --- | --- |
| Adaptive Coping (Brief-COPE) | *F*_(2, 488)_ = 0.918,  *p* = .400,  η_p_² = .004 | *F*_(1, 266)_ = 1.409,  *p* = .236,  η_p_² = .005 | Control vs. ED (Pre-test differences; p =.022) |
|  |  |  | ED vs. No ED (No pre-test differences; p =1.000) |
|  |  |  | Control vs. No ED (No pre-test differences; p = .070) |
| Maladaptive Coping (Brief-COPE) | *F*_(2, 488)_ = 1.801,  *p* = .166,  η_p_² = .007 | *F*_(1, 266)_ = 0.072,  *p* = .789,  η_p_² = .001 | Control vs. ED (No pre-test differences; p =.770) |
|  |  |  | ED vs. No ED (No pre-test differences; p =.327) |
|  |  |  | Control vs. No ED (No pre-test differences; p =1.000) |
| Instrumental Coping (Brief-COPE) | *F*_(2, 488)_ = 1.156,  *p* = .316,  η_p_² = .005 | *F*_(1, 266)_ = 0.084,  *p* = .773,  η_p_² = .001 | Control vs. ED (No pre-test differences; p =.452) |
|  |  |  | ED vs. No ED (No pre-test differences; p =1.000) |
|  |  |  | Control vs. No ED (No pre-test differences; p =1.000) |
| Anxiety (GAD-7) | *F*_(2, 494)_ = 0.289,  *p* = .749,  η_p_² = .001 | *F*_(1, 272)_ = 1.141,  *p* = .286,  η_p_² = .004 | Control vs. ED (No pre-test differences; p =.821) |
|  |  |  | ED vs. No ED (No pre-test differences; p = .716) |
|  |  |  | Control vs. No ED (Pre-test differences; p = .031) |
| Depression (PHQ-9) | *F*_(2, 448)_ = 0.487,  *p* = .615,  η_p_² = .002 | *F*_(1, 247)_ = 0.019,  *p* = .892,  η_p_² = .001 | Control vs. ED (No pre-test differences; p =.831) |
|  |  |  | ED vs. No ED (No pre-test differences; p =.922) |
|  |  |  | Control vs. No ED (No pre-test differences; p =.053) |
| Global EC (PEC) | *F*_(2, 501)_ = 4.845,  *p* = .008,  η_p_² = .019 | *F*_(1, 271)_ = 0.008,  *p* = .928,  η_p_² = .001 | Control vs. ED (Pre-test differences; p =.035) |
|  |  |  | ED vs. No ED (Pre-test differences; p =.036) |
|  |  |  | Control vs. No ED (No pre-test differences; p =1.00) |
| - Intrapersonal EC | *F*_(2, 501)_ = 5.342,  *p* = .005,  η_p_² = .021 | *F*_(1, 271)_ = 0.002,  *p* = .962,  η_p_² = .001 | Control vs. ED (No pre-test differences; p =.053) |
|  |  |  | ED vs. No ED (No pre-test differences; p =.059) |
|  |  |  | Control vs. No ED (No pre-test differences; p =.050) |
| - Interpersonal EC | *F*_(2, 501)_ = 1.845,  *p* = .159,  η_p_² = .007 | *F*_(1, 271)_ = 0.047,  *p* = .829,  η_p_² = .001 | Control vs. ED (No pre-test differences; p =.098) |
|  |  |  | ED vs. No ED (Pre-test differences; p =.044) |
|  |  |  | Control vs. No ED (No pre-test differences; p =1.000) |
| Global trait EI (TEIQue-SF) | *F*_(2, 250)_ = 1.504,  *p* = .224,  η_p_² = .012 | *F*_(1, 133)_ = 0.009,  *p* = .923,  η_p_² = .001 | Control vs. ED (No pre-test differences; p =.090) |
|  |  |  | ED vs. No ED (Pre-test differences; p =.006) |
|  |  |  | Control vs. No ED (No pre-test differences; p =.524) |
| - Emotionality | *F*_(2, 377)_ = 0.227,  *p* = .797,  η_p_² = .001 | *F*_(1, 197)_ = 0.172,  *p* = .678,  η_p_² = .001 | Control vs. ED (No pre-test differences; p =.078) |
|  |  |  | ED vs. No ED (No pre-test differences; p =.087) |
|  |  |  | Control vs. No ED (No pre-test differences; p =1.000) |
| - Self-Control | *F*_(2, 390)_ = 3.322,  *p* = .037,  η_p_² = .017 | *F*_(1, 213)_ = 0.015,  *p* = .904,  η_p_² = .001 | Control vs. ED (No pre-test differences; p =1.000) |
|  |  |  | ED vs. No ED (No pre-test differences; p =.215) |
|  |  |  | Control vs. No ED (No pre-test differences; p =.229) |
| - Sociability | *F*_(2, 381)_ = 2.634,  *p* = .073,  η_p_² = .014 | *F*_(1, 206)_ = 0.455,  *p* = .501,  η_p_² = .002 | Control vs. ED (No pre-test differences; p =.069) |
|  |  |  | ED vs. No ED (No pre-test differences; p =.070) |
|  |  |  | Control vs. No ED (No pre-test differences; p =1.000) |
| - Wellbeing | *F*_(2, 388)_ = 2.287,  *p* = .103,  η_p_² = .012 | *F*_(1, 214)_ = 0.189,  *p* = .664,  η_p_² = .001 | Control vs. ED (Pre-test differences; p =.044) |
|  |  |  | ED vs. No ED (Pre-test differences; p =.011) |
|  |  |  | Control vs. No ED (No pre-test differences; p =1.000) |
| Wellbeing (WHO-5) | *F*_(2, 493)_ = 0.252,  *p* = .777,  η_p_² = .001 | *F*_(1, 272)_ = 0.021,  *p* = .884,  η_p_² = .001 | Control vs. ED (No pre-test differences; p =1.000) |
|  |  |  | ED vs. No ED (No pre-test differences; p =1.000) |
|  |  |  | Control vs. No ED  (Pre-test differences; p = .031) |

*Note.* Age moderation refers to the Change × Group × Age interaction. Gender moderation refers to the Change × Group × Gender interaction. EC = Emotional Competencies; ED: Intervention group with Emotional Diary; GAD-7 = Generalized Anxiety Disorder scale; No ED: Intervention group without Emotional Diary; PHQ-9 = Patient Health Questionnaire; TEIQue = Trait Emotional Intelligence Questionnaire; WHO-5 = World Health Organization Well-being Index.
